# Evaluation of pharmacovigilance systems for reporting medication errors in Africa and the role of patients using a mixed-methods approach

**DOI:** 10.1371/journal.pone.0264699

**Published:** 2022-03-03

**Authors:** George Tsey Sabblah, Seth Kwaku Seaneke, Mawuli Kushitor, Florence van Hunsel, Katja Taxis, Mahama Duwiejua, Eugène van Puijenbroek

**Affiliations:** 1 Food and Drugs Authority, Accra, Ghana; 2 PharmacoTherapy, Epidemiology and Economics, Groningen Research Institute of Pharmacy, University of Groningen, Groningen, The Netherlands; 3 The Department of Health Policy Planning and Management, University of Health and Allied Sciences, Ho, Volta Region, Ghana; 4 Netherlands Pharmacovigilance Centre Lareb, ‘s-Hertogenbosch, The Netherlands; 5 School of Pharmacy, College of Health Sciences, University of Ghana, Legon, Accra, Ghana; University of Cape Town, SOUTH AFRICA

## Abstract

**Background:**

Reviewing the epidemiological profile of medication errors (MEs) reported by African countries and the systems put in place to report such errors is crucial because reporting plays an important role in improving patient safety. The objectives of this study were to characterize the profile of spontaneously reported MEs submitted by African countries to VigiBase; the World Health Organization (WHO) global database of individual case safety reports, describe systems in place for reporting these errors, and explore the challenges and facilitators for spontaneous reporting and understand the potential role of patients.

**Methods:**

In the present study, we used, a mixed-methods sequential explanatory design involving a quantitative review of ME reports over a 21-year period (1997–2018) and qualitative interviews with employees from African countries that are members of the WHO Program for International Drug Monitoring (WHO PIDM). Descriptive statistics were used to summarize key variables of interest.

**Results:**

A total of 4,205 ME reports were submitted by African countries to VigiBase representing 0.4% of all reports in the database. Only 15 countries out of the 37 WHO PIDM members from Africa contributed ME to reports, with 99% (3,874) of them reports originating from Egypt, Morocco, and South Africa. The reasons given for low reporting of MEs were weak healthcare and pharmacovigilance systems, lack of staff capacity at the national centers, illiteracy, language difficulties, and socio-cultural and religious beliefs. Some facilitators suggested by the participants to promote reporting included proactive engagement of patients regarding issues relating to MEs, leveraging on increased technology, benchmarking and mentoring by more experienced national centers. Sixteen of the twenty countries interviewed had systems for reporting MEs integrated into adverse drug reaction reporting with minimal patient involvement in seven of these countries. Patients were not involved in directly reporting MEs in the remaining 13 countries.

**Conclusions:**

MEs are rarely reported through pharmacovigilance systems in African countries with limited patient involvement. The systems are influenced by multifactorial issues some of which are not directly related to healthcare.

## Introduction

Patients living in low-income countries experience medication-related harm two or more times more frequently than those in high-income countries [1, 2]. Studies from various African countries also show high ME rates in different settings using different methods [3–5] The preponderance of MEs in low-income countries is attributed to weak medication use systems, and limited staff thereby affecting medicine prescribing, transcribing, dispensing, administration and monitoring practices [1, 2].

In March 2017, the World Health Organization (WHO) launched a global campaign dubbed the Global Patient Safety Challenge. As the third of its kind, this project aimed to obtain worldwide commitment and action to reduce medication-related harm due to weaknesses in healthcare systems by 50% in 2022 [6]. To achieve this goal, countries are required to establish functional reporting systems and involve patients in ME reporting.

Involving patients in ME reporting will likely improve reporting rate and root cause analysis leading to the prevention of these errors. The role of patients in medication error reporting is important [7] because patients are usually the first to notice any problems as a result of MEs [8]. Patient contributions to ME reporting may therefore result in the timely identification of new safety signals with medicines and other healthcare products and lead to a reduction in preventable adverse events [9].

MEs are under-reported due to fear of legal action, blaming of individuals instead of the system, lack of effective reporting systems, lack of feedback, and failure to provide support to the person who has committed the error [10–13]. Soydemir, *et al*. [14] identified barriers related to medication error reporting systems such as lack of a reporting system and lack of knowledge about a medication error reporting system.

The literature concerning MEs in African countries is relatively recent [15], with a paucity of information regarding ME reporting systems in these countries and barriers to reporting errors. A study involving ME systems in 16 countries, including three in Africa (Ghana, Zambia and Uganda) in 2012, showed that only Zambia had a ME reporting system integrated into adverse event reporting [16]. Although ME reporting systems had been mentioned as existing in Egypt [17], South Africa [18] and Morocco [19, 20], the role of patients in contributing reports to these systems was not stated.

This study aims to firstly, characterize the profile of spontaneously reported MEs submitted by African countries to VigiBase. In addition, it describes systems in place for reporting these errors, explored system challenges, and facilitators for reporting MEs, and the potential role of patients.

## Materials and methods

We used a mixed-methods sequential design in this study [21]. The quantitative study characterized the profile of spontaneously reported MEs submitted by African countries to VigiBase, whilst the qualitative study explored the systems for reporting MEs, reasons for low reporting, the current level of patient involvement, and facilitators to improve reporting. The link between the quantitative and qualitative studies was established in the way that the participants in the interviews were asked to reflect on the number of ME reports received from African countries and the systems put in place for reporting such errors.

**Fig 1:**
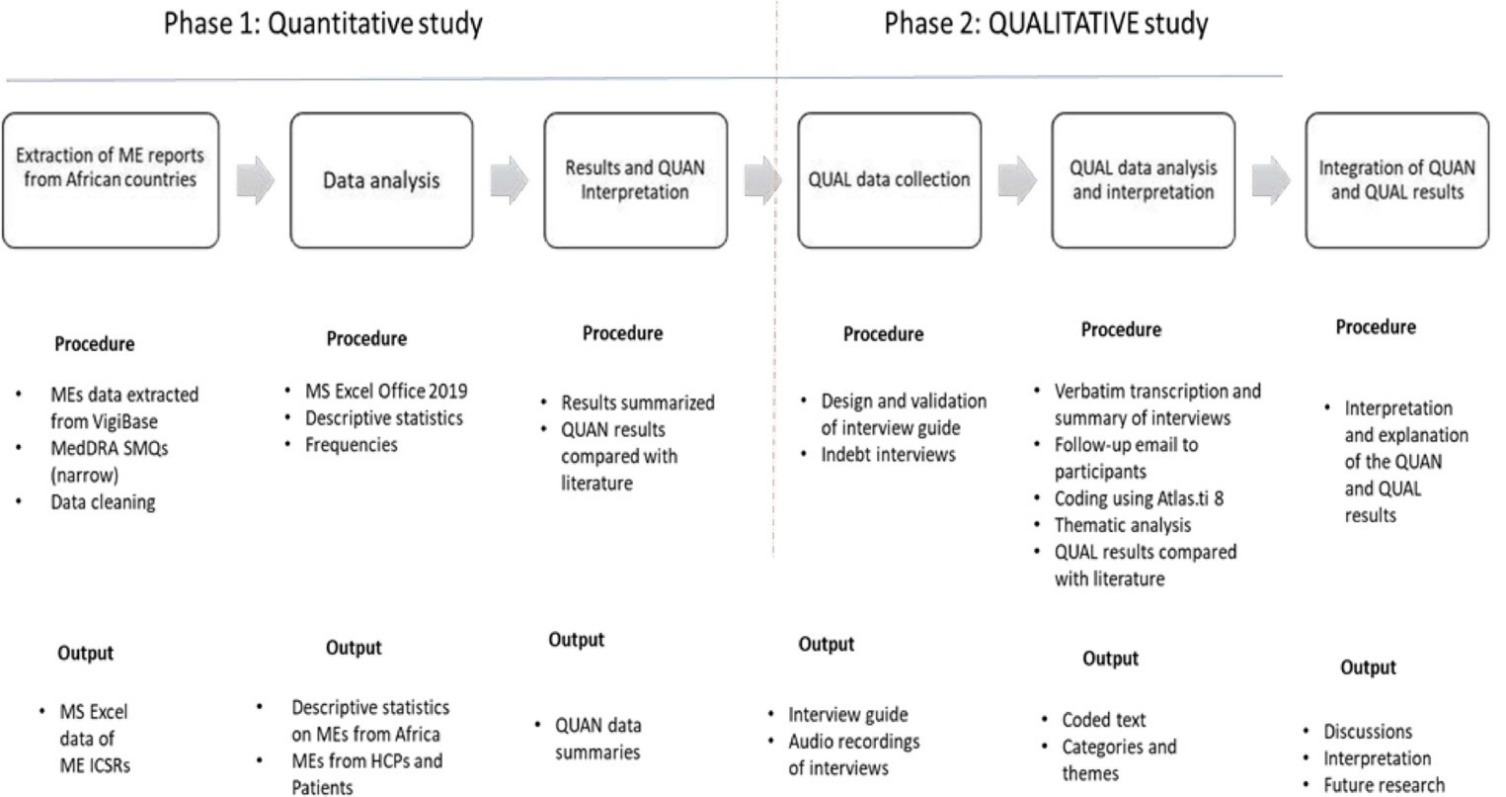
Visual model for the mixed-methods sequential explanatory study design. (QUAN: Quantitative study; QUAL: Qualitative Study; ME: medication error; MedDRA: Medical Dictionary for Drug Regulatory Authorities; SMQs: Standard MedDRA Queries; HCPs: Healthcare professionals; ICSRs: Individual Case Safety Reports).

Fig 1 shows the visual model for the mixed-methods sequential study design.

### Phase 1: Quantitative study

#### Study design and data source

This phase utilized a quantitative retrospective study design using ME reports in VigiBase from African countries who are members of the WHO Program for International Drug Monitoring (PIDM). VigiBase is the largest database which contains over 25 million anonymized individual case safety reports of suspected adverse effects of conventional and traditional medicines (herbals), as well as biological products and vaccines submitted since 1968. Currently, over 150 member countries of the WHO PIDM are actively contributing data to VigiBase. VigiBase was developed and maintained by the Uppsala Monitoring Centre (also called the WHO PIDM) on behalf of the WHO.

#### Data acquisition

Individual case safety reports (ICSRs) submitted by African countries from January 1, 1997 to December 31, 2018, in VigiBase were extracted on January 15, 2020 to MS Excel using narrow Standard MedDRA Queries (SMQs). These reports were coded at the preferred term level as MEs using MedDRA version 22.1 [22]. The narrow SMQs were used as the search criteria because they are highly likely to represent the condition of interest and therefore unlikely to introduce data not relevant to MEs. Suspected duplicate reports were automatically identified using vigiMatch, an algorithm that uses a statistical model to score pairs of reports [23, 24]. ME reports included in the analysis were obtained from healthcare professionals and patients/consumers.

#### Data analysis

Descriptive statistics were used to summarize the key variables of interest, namely, reports related to MEs submitted by each country, reported events classified at the preferred term level, year in which the reports were first submitted to VigiBase, the source of report (spontaneous or study report), the reporter type (patient or healthcare professional); and most reported medicines classified by the second-level Anatomical Therapeutic Chemical (ATC) codes.

### Phase 2: Qualitative study

#### Study design

In-depth interviews were conducted among employees from African countries that are full members of the WHO PIDM to elicit ME reporting at all levels.

#### Theoretical approach

The study employed the systems approach by von Bertalanffy [25] to review inputs (requirements) into the system for reporting MEs at three strata; patient, healthcare professional and the national center levels leading to outputs (i.e., ME reporting, prevention of MEs, increased reporting of MEs, and improved patient safety). The systems approach is based on the concept that systems cannot be reduced to a series of parts functioning in isolation, however, in order to understand the whole, one must understand the interrelations between the component parts [25]. Therefore, to implement a functional and efficient ME reporting system, one must understand the system barriers at each level of the ME reporting and how they interact with one another.

**Fig 2:**
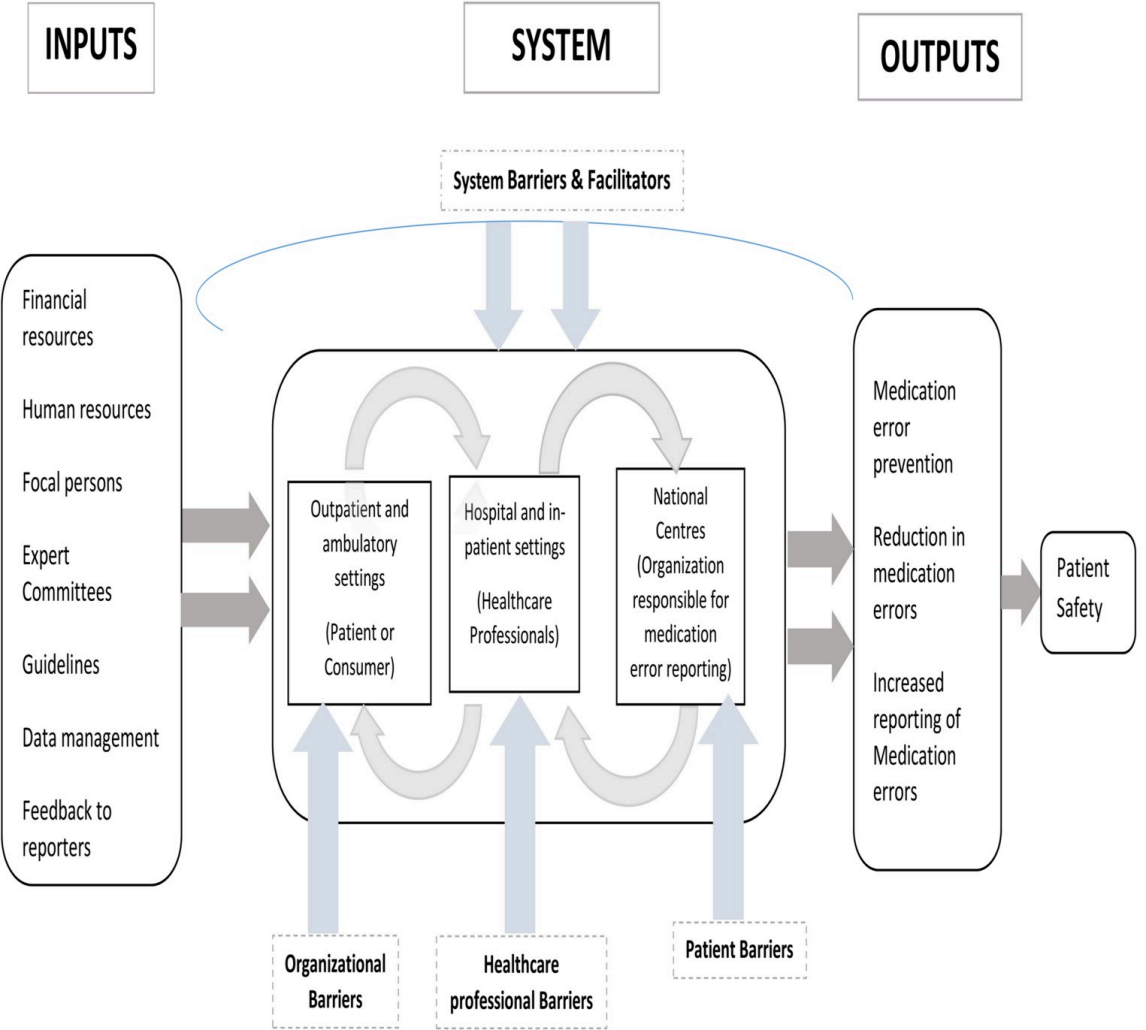
Conceptual model for the study based on the system thinking.

Fig 2 provides the theoretical framework for ME reporting based on systems thinking.

#### Study population and recruitment

The target population was made up of the heads or senior officers from all 36 African countries who are full members of the WHO PIDM. These participants have important and useful perspectives on ME reporting and systems in place in their countries. The list of contacts for the national centers was obtained from the WHO PIDM and emails sent to all of them inviting them to participate in this study. The date and time of interviews were arranged with those who agreed to participate in the study.

#### Interview guide

An interview guide was used in this study. The guide was developed based on a report of the Expert Group on Safe Medication Practices [26] and on insights from the retrospective quantitative study. Prior to this study, the guide was pretested with three pharmacovigilance experts from the national center in Ghana. The pre-testing resulted in minor changes to type and sequence of interview questions. Primarily, the questions covered by the guide are: (a) What are the characteristic features of ME reporting system in your country? (b) What are the system challenges or barriers to reporting MEs? (c) What are the facilitators for reporting MEs and (d) What is the role of patients in reporting medication errors? The resulting guide was then reviewed for content validity by the research team. The interview guide is available as S1 Appendix.

#### Data collection

The principal investigator is a male pharmacist, and responsible for pharmacovigilance in Ghana Food and Drugs Authority at the time of the study. He had obtained training in qualitative research prior to the commencement of this study. The principal investigator conducted all interviews in the English using the following electronic media with the number of participants interviewed by media type indicated: Microsoft Teams (14 participants), Zoom (3 participants), Telephone calls (2 participants) and WhatsApp call (1 participants). Each interview lasted between 20 to 45 minutes, and there were no repeat interviews.

All interviews were audio-recorded and verbatim transcription was performed by a research assistant having experience in qualitative research. The transcripts were checked for accuracy by the principal investigator. Verbatim transcription and summary of the transcripts, including the principal investigator’s interpretation of the information provided, were shared with participants for confirmation, correction, or input when necessary.

#### Data analysis

Deductive and inductive content analysis frameworks were used for analysis of the data collected with coding and analysis facilitated by use of the Atlas.ti 8 qualitative software package, Scientific Software Development GmbH, Berlin, Germany.

The deductive codes were derived from the report of the Expert Group on Safe Medication Practices [26], and the outcome of our quantitative study in phase 1. The deductive codes were developed prior to data analysis while the inductive codes were derived from the data. The deductive codes obtained were connected to relevant statements from the data obtained. The inductive codes were derived iteratively and independently by the principal investigator and co-author (MK) from the interview transcripts.

The codes were later merged, and where there were disagreements between codes, these were discussed until consensus was reached. The codes were revised iteratively, and during this process, similar codes were grouped together into sub-themes, followed by themes with a group of themes placed under categories, based on the objectives of the study. The themes and sub-themes were later discussed with the research team which was made up of all co-authors. This process involved discussions and consensus building amongst co-authors on the names of themes and sub-themes. During this process, certain themes and sub-themes were merged, and others were separated in order to clearly explain unique ideas expressed by participants.

The outcome of the qualitative study was reported in line with the checklist contained in the consolidated criteria for reporting qualitative research [27].

#### Ethical issues

Ethics approval for the study was obtained from the Committee on Human Research, Publications and Ethics, Kwame Nkrumah University of Science and Technology, School of Medical Sciences, and the Komfo Anokye Teaching Hospital; CHRPE/AP/159 /20. All study participants consented to be interviewed by responding to an email inviting them to do so. To ensure participants’ confidentiality, all identifiers were removed before data analysis. No compensation was paid to participants.

The principal investigator and co-author, SKS, are employees of the Food and Drugs Authority, Ghana. Other authors have nothing to declare.

## Results

### Quantitative study

A total of 4,205 ME reports were received from 15 African countries out of the 36 national centers in Africa within a 21-year period. However, 282 ME reports were excluded from analysis because it was not possible to determine whether these reports were from healthcare professionals or patients/consumers. Of the 3,923 reports analyzed, 2,299 (59%) were reported by healthcare professionals and the remaining 1,624 (41%) were reported by patients/consumers. Table 1 shows the 15 countries who reported to VigiBase, the year of joining the WHO PIDM, income levels based on World Bank classifications, and the number of reports submitted by healthcare professionals and patients/consumers.

**Table 1:** Number of medication error reports submitted by African countries to VigiBase, 1997–2018.

Country	Geographical Region	Year of Joining WHO PIDM*	WB Income Level**	Patient/ Consumer	Healthcare Professional	Total
Botswana	South	2009	Upper middle income		2	2
Cabo Verde	West	2012	Lower middle income	1	7	8
Egypt	North	2002	Lower middle income	780	389	1,169
Eritrea	East	2012	Low income		1	1
Ghana	West	2001	Lower middle income		2	2
Kenya	East	2010	Lower middle income		6	6
Morocco	North	1992	Lower middle income	683	1,225	1,908
Mozambique	South	2005	Low income		2	2
Namibia	South	2009	Upper middle income		3	3
Nigeria	West	2005	Lower middle income		2	2
Sierra Leone	West	2008	Low income		1	1
South Africa	South	1992	Upper middle income	160	637	797
Uganda	East	2008	Low income		7	7
Zambia	South	2010	Lower middle income		1	1
Zimbabwe	South	1998	Low income		14	14
**Total**		** **		**1,624**	**2,299**	**3,923**

* World Health Organization Program for International Drug Monitoring (WHO PIDM)

**The World Bank (WB). World Bank Country and Lending Groups [Internet]. [cited 2021 Apr 30]. Available from: https://datahelpdesk.worldbank.org/knowledgebase/articles/906519-world-bank-country-and-lending-groups

The data showed that 99% (3,874) of the ME reports came from three countries, namely, Morocco, Egypt and South Africa, with the countries recording 49% (1,908), 30% (1,169) and 20% (797), respectively.

**Fig 3:**
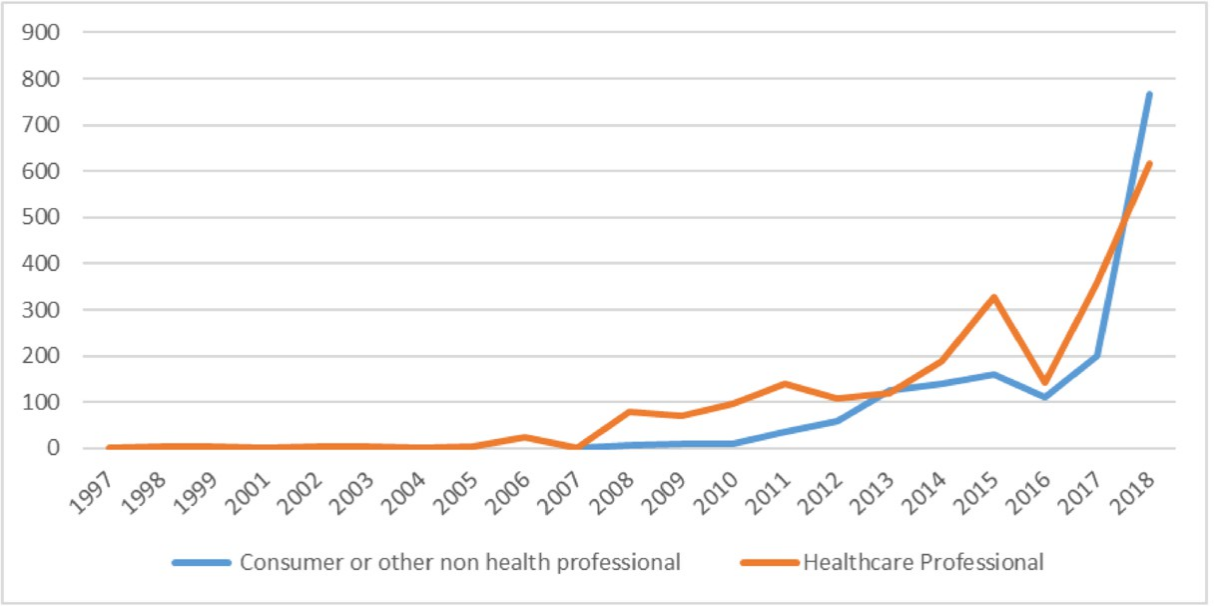
Medication error reports submitted by fifteen African countries, 1997–2018.

There was a steady increase in the number of ME reports submitted to VigiBase starting in 2007, with the maximum increase between 2016 and 2018. Fig 3 presents the number of ME reports from patients and healthcare professionals over the 21-year period.

The total number of ME reports from African countries using the standard MedDRA queries (narrow) constituted 0.4% of all ME reports in the WHO database.

**Fig 4:**
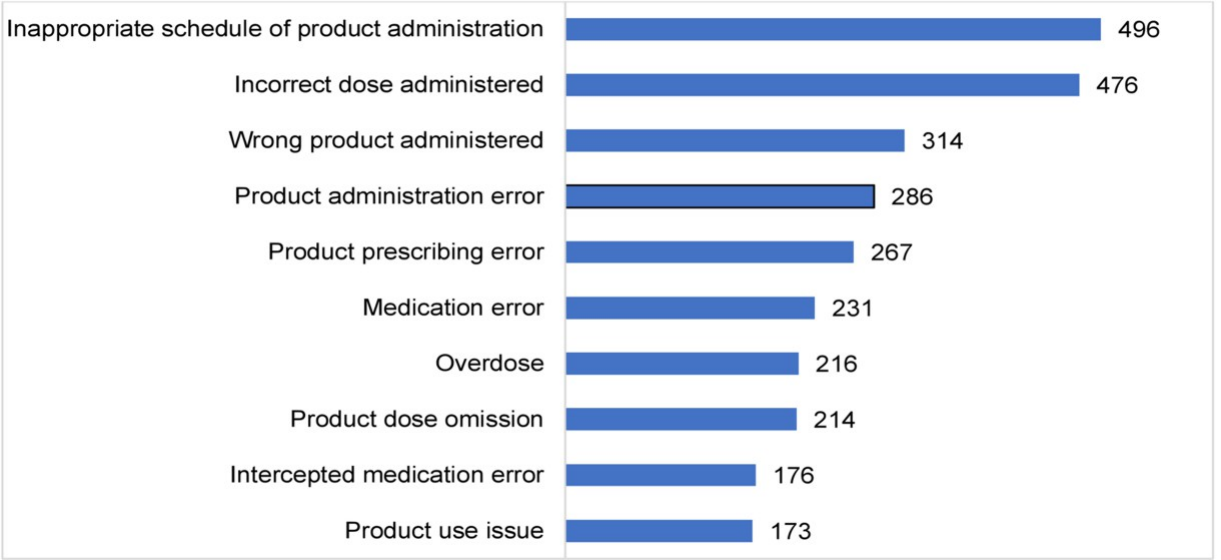
Top 10 reported medication error classes from African countries.

The top five ME classes by the MedDRA-preferred term level were; inappropriate schedule of product administration (496, 11.6%), incorrect dose administered (476, 11.1%), wrong product administered (314, 7.3%), product administration error (286, 6.7%) and product prescribing error (267, 6.2%). The top 10 ME classes from African countries is shown in Fig 4.

The study also showed that medication classes with the most commonly reported errors classified by the second- level ATC codes were drugs used to treat diabetes (949, 13.3%), antineoplastic agents (648, 9.1%), analgesics (507, 7.1%), antibacterials for systemic use (441, 6.2%), and psycholeptics (311, 4.4%). The mean age of those who experienced MEs was 29.4 years (SD± 23.8). The percentage of MEs in children younger than 17 years was 32.8% (19.8% of these were infants and children below 5 years of age and 13.0% 5–17 years); 38.9% of the MEs occurred in adults between 18 and 64 years and 8.3% in the elderly (over 65 years of age). For 20.0% of the MEs, the patient’s age was unknown.

### Qualitative study (in-depth interviews)

#### Study participants

Key informants from 20 countries who were members of the WHO PIDM took part in interviews from July 2020 to October 2020. The demographic characteristics of the participants are presented in Table 2.

**Table 2:** Demographic characteristics of participants.

Characteristics		n (%)
Gender		
	Male	14(70.0)
	Female	6(30.0)
Professional background		
	Pharmacist	16(80.0)
	Pharmacologist	1(5.0)
	Physician	1(5.0)
	Nurse	1(5.0)
	Dental surgeon	1(5.0)
Highest level of education		
	First Degree	2(10.0)
	Master’s degree	17(85.0)
	PhD	1(5.0)
Current Position		
Head of the National Centre		12(60.0)
Senior Officer		8(40.0)
Number of years worked at the National Centre		
	1–5 years	7(35.0)
	6–10 years	10(50.0)
	>10 years	3(15.0)

#### Synthesis of the qualitative findings

A total of 10 themes and 44 sub-themes emerged from the data collated data as shown in Table 3. The themes were grouped into four categories below based on the objectives of the qualitative interviews.

- description of the systems for reporting MEs,
- potential role of patients.
- challenges or barriers for reporting MEs and
- facilitators for reporting MEs

**Table 3:** Themes and sub-themes emerging from the data.

Categories	Themes	Sub-themes
Systems for reporting medication errors	Features of medication error reporting systems	1) Availability of medication error reporting system for patients
		2) The role of focal persons or drug and therapeutic committees
		3) Review of medication error reports received by expert committees or volunteers
		4) Mandatory or voluntary reporting of medication errors
		5) Anonymity and confidentiality of reporters
		6) Data management of medication error reports
		7) Tools and guidelines for reporting medication errors
		8) Feedback to reporters
	Infrastructure to support medication error reporting	1) Resources needed by national centres
		2) Resources needed by patients
Patients’ involvement in reporting medication errors	Role of patients in medication error reporting	1) Absence or minimal level of patient involvement
		2) Formal systems for patient reporting
	Attitudes to patients’ involvement	1) Positive attitude towards patients’ role
		2) Belief patients will wrongly accuse healthcare professionals
Barriers resulting in low reporting	System barriers	1) Weak healthcare and pharmacovigilance systems
		2) Lack of funding to support medication error reporting
	Organizational barriers	1) Inability to submit reports to VigiBase
		2) Lack of capacity and inadequate staff and the national centers
		3) Lack of prioritization or underestimation of medication errors
		4) Lack of feedback
	Healthcare professional barriers	1) Fear of consequences of reporting
		2) Lack of knowledge and awareness of the reporting system and procedures
		3) Lack of time and ability to diagnose medication errors
	Patient barriers	1) Fear of reprisal from healthcare professionals
		2) Lack of knowledge by patients
		3) Illiteracy and language difficulties
		4) Socio-cultural and religious beliefs
Facilitators for reporting	Collaboration with stakeholders	1) Proactively engage patients
		2) Harmonize efforts on the continent
		3) Benchmarking and mentoring
	Strengthening structures for reporting	1) Include medication error reporting in existing learning programs
		2) Leverage on increased technology
		3) Review legislation to include medication error reporting

The S1 Table shows the themes and sub-themes emerging from the interviews with illustrative verbatim examples of unique ideas expressed by individual participants.

*Systems for reporting MEs*. The results indicated that three (15%) countries, namely, Liberia, Malawi, and Burkina Faso have no systems for ME reporting. One (5%) country, Kenya, had included ME reporting in the guidelines on safety and vigilance of medical products and health technologies launched 6 months prior to the interview. The remaining 16 (80%) countries had some systems in place for reporting MEs. ME reporting in the 16 countries with systems was grouped under two themes; features of the systems and infrastructure needed to support them are outlined below.

- The ME reporting function is coordinated at a national level by the National Medicine Regulatory Authorities (NMRAs) in 14 (87.5%) of the 16 countries with a system for reporting MEs except in two, namely Morocco and the Democratic Republic of Congo. In these two countries, management of the ME reports is by the national centers and regulatory action based on reports received are referred to the NMRAs for regulatory decision and action.
- Six (37.5%) of the 16 countries with systems for reporting MEs mentioned the presence of focal persons (3) or drug and therapeutic committees (3) for ME reporting at the facility level. The role of these focal persons at the facility level was to act as a link between the national center and the facilities, collate ME reports, and carry out analysis at the facility level before submitting reports to the national center.
- Medication reporting is voluntary in 15 (93.8%) out of the 16 countries, while mandatory reporting was recorded in only one (6.2%) country: Namibia.
- Four (25%) of the 16 countries mentioned the existence of expert committees or medical volunteers that are responsible for reviewing ME reports and making recommendations for regulatory action.
- Three (18.8%) countries mentioned the use of the preventability (P) method to identify ME reports from other ICSR reports received. The P method is a standardized instrument developed by the WHO with 20 explicit criteria for assessing the preventability of adverse drug reactions [28]. Participant 07 (PA07) expounded that: *“*This is how we get mainly from patients and regarding MEs, sometimes we also identify them using the preventability method that we use in our center, the P method.”
- Assessing the presence of tools and guidelines for reporting MEs, 6 (37.5%) of the 16 countries had ME reporting mentioned in their existing guidelines, whereas 7 (43.8%) had no guidelines for reporting MEs. PA08 explained that: “Okay, we don’t have a guideline till now we don’t have a guideline we still using a form, eh but we don’t have a special form till now; we don’t have a guideline ….”
- In terms of the infrastructure needed to support existing ME reporting systems, participants suggested financial resources (44%), technical resources (27%), and human resources (13%). The rest were electronic reporting tools (10%), databases (3%), and political will (3%).

*Patient involvement in reporting MEs*. Patient involvement in reporting MEs was captured under two themes: the role of patients in ME reporting, and attitudes toward patient involvement.

Participants from seven countries (43.8%) mentioned that there were formal systems for patients to play a role in reporting MEs. Of the seven countries, six stated that the national centers were not the preferred choice for patients to send their reports; they would rather report to healthcare professionals or the media. For instance, PA19 stated that “We are not usually the first point of communication to whom patients come to immediately when they are affected with some kind of ME.” Only one participant (PA07) stated that patients report directly to the national center in her country mainly through telephone calls with a number of signals from reports received from patients. She explained “I think 16 signals since we put in place this unit and they have contributed to maybe half of the signals”

Seventeen (85%) participants expressed positive opinions regarding patient involvement in ME reporting. Those who expressed positive opinions believed that patient involvement in ME reporting would add value to the healthcare delivery system in general. They also asserted that patient involvement would make healthcare delivery complete because patients would be actively involved in their own care.

In contrast, three participants (15%) believed that this should be done cautiously because patients lack basic knowledge of what constitutes MEs and they will sometimes accuse healthcare professionals of administering the wrong medications if they are involved in ME reporting. PA08 cautioned that “We need to have it in an appropriate manner because they could transform to say that doctors are committing a lot of problems that they could be victim of that.”

*Barriers resulting in low reporting of MEs*. Four barriers identified as resulting in low reporting of MEs were system, organizational, healthcare professional, or patient-related factors.

System-related barriers identified were weak healthcare and pharmacovigilance systems, lack of funding to support ME reporting, and inability of national centers to submit reports to VigiBase. First, participants complained that in most African countries, pharmacovigilance systems are weak and at the early stages of development, they are also not decentralized, nor are their legal bases or guidelines for ME reporting. As explained by PA06 for example, “The concept of MEs is new and people don’t know much about it, even myself, it was when I went to the national center in Morocco in 2016 that I was exposed to it.”

The second system-related barrier involved lack of funding to support ME reporting. Participants stated that finances were needed to create awareness as well as to provide training on ME reporting for national center staff and healthcare professionals.

Organizational barriers identified were lack of capacity and inadequate staffing levels at the national centers, underestimation or lack of prioritization of ME issues, and lack of feedback. Participants explained that inadequate staffing levels or lack of capacity at the national centers to appropriately code ICSRs has an effect on the number of reports committed to VigiBase as ME reports. For instance, PA02 complained that “Many a times we send in reports and those reports some of those reports are actually ME reports, but then we are sending them to VigiBase as an adverse drug reaction so for me I think those are the key issues”.

Healthcare professional barriers adduced by participants were fear of consequences of reporting, lack of knowledge and awareness of the reporting system or procedures, and lack of time and ability to diagnose MEs. Fears described ranged from persecution by colleagues to fear of blame by superiors/politicians in case relatives of influential persons in the community suffered from MEs.

Participants were concerned that patients may not want to report MEs because of fear of reprisal from healthcare professionals. For instance, PA03 was worried that “Some of them fear of victimization, they think if they report this error to that facility, next time they go there, they will not get proper care, so they prefer just not to go back to that facility.”

Other patient-related barriers identified by participants were lack of knowledge by patients, illiteracy or language difficulties, and socio-cultural and religious beliefs. Regarding socio-cultural and religious beliefs, respondents explained that in most countries, males play a dominant role, and in cases where a female colleague committed an error, this may not be reported because of potential accusations by male colleagues. PA15 stated that “People, maybe men in the family, if you tell him that you have made the kind of ME, she will be blamed by her husband.” Additionally, participants mentioned that most Africans believe that things happen because God allows it, so they will not report any harm suffered from ME because of the belief that it is an Act of God. PA05 acknowledged that “I think in Africa a lot of people think that everything happens because God accepts it.”

Illiteracy or language barriers were also identified as barriers to the low reporting of MEs by patients. Participants mentioned that in some of their countries, there were different languages, and it would be difficult for the national center to design or translate the reporting forms to accommodate all such languages. Moreover, some terminologies for MEs or other medicine safety terminologies are lacking in many local languages.

*Facilitators for reporting*. Facilitators for reporting MEs identified by participants included collaboration with stakeholders and strengthening structures for reporting.

Participants recommended collaboration with stakeholders, including relevant institutions, activists, and advocators for ME reporting. Harmonization of efforts in sub-regions, in particular by regulators, to ensure ME reporting by patients is brought to the fore was also highly recommended.

Facilitators suggested that strengthening structures for reporting included ME reporting in existing learning programs, leveraging increased technology and legal amendments to protect persons who report MEs.

## Discussion

This study described the systems for reporting MEs in Africa and the characteristics of spontaneously reported MEs submitted by African countries to VigiBase. It also explored systemic barriers and facilitators for reporting MEs and the potential role of patient involvements.

### Characteristics of spontaneously reported MEs from African countries

A total of 4,205 ME reports from African countries (representing 0.4% of all ME reports in VigiBase) were submitted by African countries over a 21-year period. Only 15 of the 37 full members of the WHO PIDM contributed ME reports to VigiBase, with 99% (3,874) of these reports originating from three countries, namely, Egypt, Morocco, and South Africa.

The high reporting rates of Egypt and Morocco compared to other countries is partly because these countries have implemented special programs to promote ME reporting as part of their pharmacovigilance systems [17, 19, 29]. The Moroccan Pharmacovigilance Center and the World Alliance for Patient Safety, in collaboration with the Uppsala Monitoring Centre, piloted a program to detect MEs for which the Center acted as the project coordinator [20].

The increase in the number of reports submitted from 2016 to 2018 could be attributed to several factors. Some of these factors are the publication of the WHO Guideline on Reporting and Learning Systems for Medications Errors: the Role of Pharmacovigilance Centers in 2014 [30], implementation of the Monitoring Medicines Project between 2009 and 2013 [29], and changes to the MedDRA terminology. The WHO Guidelines for reporting MEs were developed as part of the Monitoring Medicines Project and are expected to increase the capacity of national centers to analyze reports of MEs, identify preventable MEs, and support actions to minimize the occurrence of preventable MEs [31].

Medication classes with the most commonly reported errors classified by second-level ATC codes were drugs employed in treating diabetes, antineoplastic agents, analgesics, antibacterials for systemic use, and psycholeptics. The results of our study are comparable to those of Alj et al. [32].

### System for reporting MEs

ME reporting systems in African countries generally complied with the characteristics of effective ME reporting systems, which are independent of regulatory or accrediting processes for healthcare professionals, provision of opportunity for evaluating the causes of errors, non-punitive approaches to reporting, and provision of feedback on the analysis of error results to those involved in ME reporting. Others include the presence of guidelines, anonymity, confidentiality, and voluntariness of reporting [1, 2].

First, ME reporting in African countries was generally hosted by NMRAs with responsibility for data collection, analysis, feedback, and regulatory action, except in two countries, namely Morocco and the Democratic Republic of Congo, where the NMRA was not directly responsible for coordinating these processes.

Second, the system for reporting MEs was described as voluntary in all countries with systems for reporting except one, where ME reporting was mandatory for healthcare professionals. Therefore, it is important for national centers to highlight the voluntary and non-punitive nature of reporting systems in order to reduce the fear of reporting expressed by a majority of participants.

The third feature of ME reporting systems in Africa was the absence of guidelines or information regarding MEs in the existing pharmacovigilance guidelines in most countries investigated. A ME reporting system without guidelines cannot comply with acceptable international standards. Such countries need to take steps to update existing policies and guidelines to include ME reporting. These guidelines will help provide information concerning reportable MEs and other issues pertinent to reporting.

In the fourth instance, we noted the presence of expert committees or volunteers to assist some of the national centers to review the ME reports received. Such committees or volunteers provide opportunities for countries to evaluate reports and conduct causality assessments before feedback is provided to reporters.

The final characteristic identified was the provision of feedback; this was mentioned by all 16 countries with systems for reporting MEs. According to several studies, appropriate feedback systems improve reporting [33–36].

Availability of resources, including financial, technical, human, database, and electronic reporting tools, were highlighted as being inadequate in most countries. Some authors have suggested that African countries are at the early stages of developing pharmacovigilance systems, and have limited financial and human resources [37–40]. Our findings, therefore, reinforce the existing literature, given the number of country representatives who mentioned challenges involving the adequacy of financial and human resources.

### Barriers and facilitators for reporting MEs

Some of the barriers outlined by participants as reasons for the low reporting rates of MEs have been widely studied. Some fear the consequences of reporting [11, 41–44], lack of knowledge and awareness of the reporting system and procedures [43, 44], lack of feedback [44], lack of time [35], and inability to diagnose MEs. Participants also attributed low rates of reporting ME to a lack of prioritization of ME reporting across the African continent.

We also found that socio-cultural and religious beliefs, political persecution, fear of victimization by healthcare workers, illiteracy, and language difficulties were identified as barriers to patient reporting of MEs. The role of religious and cultural beliefs in health-seeking behavior and health utilization has previously been documented in some African countries [45, 46], but not in relation to ME reporting.

ME reporting is influenced by factors, such as healthcare delivery systems, illiteracy, language difficulties, and socio-cultural and religious beliefs, which directly or indirectly influence patient safety. Our findings, therefore, support the use of systems theory as a framework for this study because our results are consistent with the tenets of such systems approaches, which postulate that a system is greater than the sum of its parts, and that these parts are interdependent and interact through mutual feedback procedures such that changes to one part of the system directly or indirectly influence other parts.

### Potential role of patients

Patient involvement in ME reporting in Africa is minimal, and participants believe that involving patients will be a ‘game changer’ for ME reporting on the continent since this will not only improve ME reporting, but will also add value to healthcare delivery systems in general. Our findings are consistent with earlier studies that found a minimal level of patient involvement in pharmacovigilance in developing countries [47]. Patient participation in ME reporting, particularly in Africa where healthcare delivery systems are weak with a high level of self-medication, will help in obtaining data regarding MEs that occur external to formal healthcare systems, which may help in signal generation.

### Study strengths and weaknesses

The strengths of our study are, first, combining quantitative and qualitative methods within a single study to explain the findings; in particular, the contributory factors for low reporting of MEs in Africa were supported by actual, real-world data regarding the number and nature of ME reports.

Second, the large number (20) of country representatives interviewed provided a clear, broad view of many aspects of the systems for ME reporting and challenges existing in Africa, including cultural and religious spheres.

Lastly, African country representatives interviewed are passionate about patient involvement in ME reporting, which is good news for pharmacovigilance across the continent.

Weaknesses of our study are, first of all, the risk of inappropriate coding of ICSRs. It is likely that the number of ME reports retrieved from VigiBase is an underestimation of the actual number of cases because the majority of these reports may not be coded as MEs. This weakness was outlined by PA02 when he complained that “Many a times we send in reports and those reports some of those reports are actually ME reports, but then we are sending them to VigiBase as an adverse drug reaction so for me I think those are the key issues”. Also, the authors retrieved only ICSRs coded as preferred term ‘medication errors’ under which cases with low level terms such as ‘inappropriate schedule of product administration, incorrect dose administered, wrong product administered, product administration error, and product prescribing error’ were captured. This possibly underestimates the number of MEs reported as there might be MEs coded with other reaction terms possibly due to inadequate knowledge in coding reaction terms. In addition, the relative low number of reports from Africa (0.4%) is also suggestive for the existence underreporting of medication errors.

Secondly, not all those approached participated in the study so these countries are not represented in the qualitative analyses.

## Conclusions

MEs are rarely reported through pharmacovigilance systems in African countries and there is limited involvement of patients in the systems for reporting MEs. Our study showed that the reasons for this limited participation are probably linked to multifactorial issues, some of which are not directly related to healthcare, and which include illiteracy, language difficulties, and socio-cultural and religious beliefs. To improve ME reporting, it is recommended that studies be conducted regarding the frequency and types of MEs reported by patients in outpatient settings and the economic and health impact of MEs in Africa. The results of such studies should be used as advocacy tools to secure buy-in by policymakers and political leaders to invest in strengthen the systems for reporting MEs, including patient involvement.

## Supporting information

S1 Table Themes and sub-themes emerging from the interviews with illustrative verbatim examples by interviewees. (DOCX)

S1 Appendix Interview guide. (DOCX)
