# Supplementary material for: Evaluation of pharmacovigilance systems for reporting medication errors in Africa and the role of patients using a mixed-methods approach
Source: PLoS One. 2022 Mar 3;17(3):e0264699. doi: 10.1371/journal.pone.0264699 (PMC8893697; doi:10.1371/journal.pone.0264699)
Supplement: S1 Table — (DOCX) [file pone.0264699.s001.docx]

**S1 Table****.** Themes and sub-themes emerging from the interviews with illustrative verbatim examples by interviewees

| **Themes and sub-themes** | **Verbatim examples of participants** |
| --- | --- |
| **Theme 1**: Features of medication error reporting systems |  |
| - 1. Availability of medication error reporting system | There are no structures for now, we work on adverse drug reactions and we don’t have anything yet for medication errors. (PA06)  I think at the moment we do not have that system specifically for medication errors or but we do receive reports of adverse reactions that maybe arisen because of medication errors. |
| - 1. The role of focal persons or   drug and therapeutic committees | In hospitals we have what we call Drug and Therapeutics Committee those are dedicated committee for medicines safety and also in private also they are involved in supply chain issues of medical products at the level of hospital. But that committee has a subcommittee called Pharmacovigilance Subcommittee that deals with adverse events, analyzing them before submitting to the center, they receive also report on medication errors. (PA15) |
| - 1. Review of medication error   reports received by expert committee or volunteers | Yeah, so what happens is that we prepare case reports for the committee review and then the committee makes the analysis and they confirm that this was a medication error, we use that report to facilitate training people for what was the cause of the error and how we can prevent such errors in the future. (PA17) |
| - 1. Mandatory or voluntary reporting of medication errors | According to our law, according to the [Law establishing the NMRA], it is mandatory for every healthcare professional to report a medication error once they have identified it, so then that’s why they have to make use of the medication error notification form………(PA14) |
| - 1. Anonymity and confidentiality of reporters | Unless you ensure confidentiality and there is insecurity in the system it is very challenging to receive report of medication errors, so, in our pharmacovigilance policy, it is clearly stipulated that all individual case safety report submitted at our pharmacovigilance center are not qualified for admission in the court of law and also they are handled with strict confidence and we can’t leave healthcare professionals, in order to build their confidence and they ensure you now reporting of medication errors without any hesitation. (PA16) |
| - 1. Data management of medication error reports | First of all, for medication error reports we use Excel spreadsheet because we still receive very few reports so at this moment there is actually no need for dedicated database because it is easy to analyze, is easy to retrieve the information because we receive very few reports. (PA10) |
| - 1. Tools and guidelines for reporting medication errors | We have the online portal, which is also one for patients and one for healthcare professionals. Then recently we have an app which is the Med Safety App, that we use to report adverse drug reaction so that one it is a single app that can be used by both healthcare professionals and patients even though it was mainly to help patient report adverse drug reactions. (PA20) |
| - 1. Feedback to reporters | Yes, we provide feedback, so when we receive the report, we have a committee known as the Pharmacovigilance Clinical Trials Committee so the committee meet once every month. The Committee will then discuss all the pharmacovigilance issues; all the clinical trials issues that are there, then the Committee makes the decision though we serve as the Secretariat to that Committee. (PA10) |
| **Themes and sub-themes** | **Verbatim examples of participants** |
| **Theme 2:** Infrastructure to support medication error reporting |  |
| 2.1 Resources needed by national centers | We need financial support and technical support in developing those tools which can be used by patients to report medication errors*.* (PA15)  Again, this is important and then again looking at it, you see this is extra work on top of work. So, again personnel and I think this is one of the biggest challenges because every time you talk to your colleagues within the continent, you realize that everyone is complaining the work is building up by the number of personnel is lacking. (PA03) |
| 2.2 Resources needed by patients | I think the awareness, creating awareness for the patients is the most important aspect is to increase the number of medication error reporting. (P10)  Another thing that it will strengthen that telephone application or the telephone net call where they can call on toll-free number to report any medication error they have in the treatment or well-being at home. (PA15) |
| **Theme 3:** Role of patients in medication error reporting |  |
| 3.1 Absence or minimal level of patient involvement | No, no patients are not yet involved even with adverse event the population, they are going to be involved now with the med safety program that Uppsala is launching. And we know that at that time we will receive reports from the population also. (PA08) |
| 3.2 Formal systems for patient reporting | Okay, for us they play a major role because we detected, I think 16 signals since we put in place this unit and they have contributed to maybe half of the signals. Patients are the source of these signals, so for us they play a major role in detecting signals regarding medication errors. (PA07) |
| **Theme 4:** Attitudes to patients’ involvement |  |
| 4.1 Positive attitude towards patients’ role | It is highly recommended that patients report medication errors, this helps us, as I already said because these are contributing factors that due to medication errors that are specific for outpatients because in hospitals pharmacists they are more working on clinical and hospital pharmacy and they are reporting to us, but in outpatients at the houses medication errors occurs and we should know about these medication errors, why they report in order to prevent them, so for us it is very important. We can have information about OTC drugs and why there is no adherence to some drugs about packaging problems and maybe this year we had a patient that call us to say that there is a difference between translation about posology…. (PA17) |
| 4.2 Belief patients will wrongly accuse healthcare professionals | We need to have it in an appropriate manner because they could transform to say that doctors are committing a lot of problem that they could be victim of that. (PA08) |
| **Themes and sub-themes** | **Verbatim examples of participants** |
| **Theme 5:** System barriers |  |
| 5.1 Weak healthcare and pharmacovigilance systems | The concept of medication errors is new and people don’t know much about it, even myself, it was when I went to the national center in Morocco in 2016 that I was exposed to it. (PA06) |
| 5.2 Lack of funding to support medication error reporting | So, if we want to introduce this first of all, like we spoke about is the platform to report this there is cost involved sending text messaging might cost them some money to send the text message, they are going to call is still going to cost some money you know, and not everyone has access again to all these platforms. Majority of the patients you know will not be able to do this with ease so that could be the cost. (PA09) |
| **Theme 6:** Organizational barriers |  |
| 6.1 Inability to submit reports to VigiBase | So, am thinking we are not there, so in terms of VigiBase, maybe our resources, we don’t have enough resources because VigiBase has financial implications as well. So maybe we ‘ve not reached that level where we are able to send our reports to VigiBase. (*PA*19) |
| 6.2 Lack of capacity and inadequate staff and the national centers | So, now like we have just about in a whole department for PV and clinical trial even though our focus is on PV and but we have two major areas combined, pharmacovigilance and clinical trial which we have six staff, six staff that are full time. (PA12)  Many a times we send in reports in those reports some of those reports are actually medication error reports, but then we are sending them to VigiBase as adverse drug reaction so for me I think those are the key issues. (PA02) |
| 6.3 Lack of prioritization or underestimation of medication errors | I can say that they underestimate the problem, the magnitude of the problem and its consequences so that maybe they are not well trained on those medication error issues so that may be mostly on reporting on adverse events, but on medication errors they are not investing a lot in training people or patronizing or reporting or managing, preventing those medication issues. (PA15) |
| 6.4 Lack of feedback | Another concern is feedback, people you know will not report if you don’t provide them with feedback, they will be discouraged and stop reporting.” (PA18) |
| **Theme 7:** Healthcare professional barriers |  |
| 7.1 Fear of consequences of reporting | The problem that we are facing is the fact that medical doctors, they are afraid to notify medication error because they see that the country is not… everything is politicized so if anyone died because it was medication error from these personnel and this guy could be persecuted. (PA08) |
| 7.2 Lack of knowledge and awareness of the reporting system and procedures | May be the only challenge is that, some of the health care workers are not aware how to report and some sort we and our information is not well disseminated in the facility. PA17 |
| 7.3 Lack of time and ability to diagnose medication errors | Most of the time healthcare professionals are very busy, they don’t even have time to actually give patients time to report. Sometimes the queue is long so I think that could also be a barrier. (PA19) |
| **Themes and sub-themes** | **Verbatim examples of participants** |
| **Theme 8:** Patient barriers |  |
| 8.1 Fear of reprisal from healthcare professionals | Some of them fear of victimization, they think if they report this error to that facility, next time they go there, they will not get proper care, so they prefer just not to go back to that facility. PA03 |
| 8.2 Lack of knowledge by patients | Basically, they don’t know they need to report medication errors. I think basically they don’t know and then also they cannot identify it”. (PA20) |
| 8.3 Illiteracy and language difficulties | The other barrier will be the language barrier because we cannot have a reporting form in every language in the country. We may choose one or two languages and the, if the patient does not understand it that will be an issue. (PA17) |
| 8.4 Socio-cultural and religious beliefs | People, maybe men in the family, if you tell him that you have made the kind of medication error she will be blamed by her husband. (PA15) |
| **Theme 9:** Collaboration with stakeholders |  |
| 9.1 Proactively engage patients | I think we have to paint the picture that the patient is the primary person and their safety should remain our key focus. (PA14)  Relevant institutions including the WHO should work extensively on advocating establishment of medication errors especially on the detection, reporting and investigation of medication errors. In this case we really need activists or advocators for establishment of such kind of systems…. (PA16) |
| 9.2 Harmonize efforts on the continent | The different health authorities around Africa need to come together and decided what can we actually do or each region in Africa need to decide what can we do and how we can harmonize our efforts and really have a stronger and a united front when it comes to saving lives because it extends beyond what we do on a daily basis but what we do on a daily basis is the most important as well. (PA14) |
| 9.3 Benchmarking and mentoring | I am particularly interested in how other countries do coordinate the healthcare system or hospitals and staff with the national health center in terms of getting this medication error report. (PA01) |
| **Theme 10:** Strengthen structures for reporting |  |
| 10.1 Include medication error reporting in existing learning program | So, when we do our sensitization, we try our best, to have time we discuss it with them and I try to inform them that reporting does not have anything to do with your competency. (PA04) |
| 10.2 Leverage on increased technology | Also, we have also decided that in this day and era because we have got a lot of our pharmacists working in other health facilities around the country, the easiest way to get a hold of them would be via electronic means but specifically on WhatsApp. So, we have established national WhatsApp groups for the healthcare professionals around the country inclusive of pharmacists. (PA14) |
| 10.3 Review legislation to include medication | I have heard that in one country in Europe the problem of medication error has been seated in the law and is now in the regulation. I don’t know if this also possible in our country because here every problem is a politics so is very difficult. (PA08) |
