# Supplementary material for: Evaluation of pharmacovigilance systems for reporting medication errors in Africa and the role of patients using a mixed-methods approach
Source: PLoS One. 2022 Mar 3;17(3):e0264699. doi: 10.1371/journal.pone.0264699 (PMC8893697; doi:10.1371/journal.pone.0264699)
Supplement: S1 Appendix — (DOCX) [file pone.0264699.s002.docx]

**S1 Appendix.** Interview Guide

**Study title:** Evaluation of pharmacovigilance systems for reporting medication errors in Africa: What is the level of patients’ involvement?

1. First, I would like to know about your:
   1. professional background,
   2. the highest educational level attained,
   3. number of years worked at the national centre,
   4. your current position and
   5. working experience at the national centre.
2. Could you please tell me about the systems and structures for reporting medication errors in your country? *[provide information on the organization responsible for medication error reporting at all levels, availability of reporting form and guidelines for reporting medication errors, data management and feedback]*
3. Could you tell me whether healthcare professionals (HCPs) are involved in medication error reporting in your country and the role they play?
4. Are patients in anyway also involved in medication error reporting? If YES, what role do they play in reporting medication errors?
5. If patients are asked to report medication errors, what type of tools and resources do you think will be needed for them to report?
6. What resources (financial and technical) and tools in your view are needed to establish or maintain a medication error reporting system for patients?
7. Please, why do you think patients should or should not be asked to contribute to medication error reporting in your country?
8. My recent analysis of medication error reports in VigiBase, indicated that out of every 1,000-medication error report submitted by countries across the world, African countries contributed only one. What do you think may be the reasons for the low reporting of medication errors by African countries?

What in your view might be the reasons for the low reporting of medication errors by patients?

1. Could you please tell me your views about what are or will be barriers to direct patient reporting of medication errors?
2. Please, are there any other issues you think we haven’t discussed which will contribute to establishment or maintenance of efficient medication error reporting system for patients?
